# 18S Ribosomal RNA Evaluation as Preanalytical Quality Control for Animal DNA

**DOI:** 10.1155/2016/6104323

**Published:** 2016-09-08

**Authors:** Cory Ann Leonard, Marina L. Meli, Marilisa Novacco, Nicole Borel

**Affiliations:** ^1^Department of Pathobiology, Institute of Veterinary Pathology, University of Zurich, Winterthurerstrasse 268, 8057 Zurich, Switzerland; ^2^Center for Clinical Studies, Vetsuisse Faculty, University of Zurich, Winterthurerstrasse 260, 8057 Zurich, Switzerland; ^3^Clinical Laboratory, Vetsuisse Faculty, University of Zurich, Winterthurerstrasse 260, 8057 Zurich, Switzerland

## Abstract

The 18S ribosomal RNA (rRNA) gene is present in all eukaryotic cells. In this study, we evaluated the use of this gene to verify the presence of PCR-amplifiable host (animal) DNA as an indicator of sufficient sample quality for quantitative real-time PCR (qPCR) analysis. We compared (i) samples from various animal species, tissues, and sample types, including swabs; (ii) multiple DNA extraction methods; and (iii) both fresh and formalin-fixed paraffin-embedded (FFPE) samples. Results showed that 18S ribosomal RNA gene amplification was possible from all tissue samples evaluated, including avian, reptile, and FFPE samples and most swab samples. A single swine rectal swab, which showed sufficient DNA quantity and the demonstrated lack of PCR inhibitors, nonetheless was negative by 18S qPCR. Such a sample specifically illustrates the improvement of determination of sample integrity afforded by inclusion of 18S rRNA gene qPCR analysis in addition to spectrophotometric analysis and the use of internal controls for PCR inhibition. Other possible applications for the described 18S rRNA qPCR are preselection of optimal tissue specimens for studies or preliminary screening of archived samples prior to acceptance for biobanking projects.

## 1. Introduction

In veterinary and human medicine, diagnostic polymerase chain reaction (PCR) and real-time quantitative PCR (qPCR) for identification of microorganisms are routinely carried out on a broad range of sample types. Furthermore, veterinary samples specifically are collected from a wide range of animal species. Fresh or frozen tissue samples are ideal for DNA extraction, amplification, and various downstream diagnostic and experimental analyses. However, diagnoses using samples, such as swabs, which may contain insufficient amounts of sample material or autolytic tissues due to delayed sampling and transport or storage under suboptimal conditions, is sometimes unavoidable. Additionally, retrospective analyses are often restricted to formalin-fixed and paraffin-embedded (FFPE) archived material. Unfortunately, DNA extracted from FFPE samples has an increased degree of degradation compared to samples generated from fresh material, resulting in reduced amplification of DNA targets exceeding 200 base pairs (bp) [1].

The goal of this study was to evaluate samples collected during routine pathological examination and/or Chlamydiaceae family-specific qPCR analysis for the presence of the eukaryotic 18S ribosomal RNA (rRNA) gene, with the aim of determining the presence of PCR-amplifiable host DNA as an indicator of sufficient sample quality for qPCR analysis. The primary intended application of this type of analysis is the differentiation of samples with DNA quality/quantity sufficient for microbial identification from those samples with degraded DNA or insufficient sample size/content. This can serve to help identify false negative samples in diagnostic and research settings and/or to screen samples for appropriate DNA quantity/quality before subsequent evaluation. Another possible application for the described 18S rRNA qPCR is preliminary screening of archived samples prior to acceptance for biobanking projects.

## 2. Materials and Methods

### 2.1. Formalin Fixation and Paraffin Embedding

Samples were collected immediately after necropsy and fixed in 10% formalin (e.g., 4% formaldehyde) for 24 to 48 hours. Formalin-fixed liver samples were embedded in paraffin and processed as previously described [2].

### 2.2. DNA Extraction

DNA was manually extracted from fresh tissues and swabs using a DNA extraction kit with spin-column format (DNeasy Blood and Tissue Kit, Qiagen AG, Hilden, Germany) or automated equipment and associated extraction kit, which uses paramagnetic bead separation (Maxwell 16 MDx Instrument and Maxwell 16 Tissue DNA Purification Kit, Promega Corporation, Madison, WI), per manufacturer's instructions.

For FFPE samples, 20–30 *μ*m sections of samples were deparaffinized in xylene and centrifuged at 13,500 ×g for 5 min. Subsequently, xylene was removed by ethanol extraction followed by the removal of ethanol. The resulting deparaffinized pellet was treated with proteinase K and incubated overnight on a thermomixer set to 55°C and agitated at 550 rpm. The DNA was then extracted manually or using the automated instrument/kit.

To generate DNA from HeLa cells (human cervical adenocarcinoma epithelial cells, CCL-2, American Type Culture Collection, Manassas, VA), 4 × 10^6^ cells/mL were suspended in sterile phosphate buffered saline prior to subsequent manual and automated DNA extraction. 300 *μ*L cell suspension and elution volumes were used for automated extraction and 200 *μ*L cell suspension and elution volumes for manual extraction, in accordance with manufacturer's recommendations, to yield DNA corresponding to 4 × 10^3^ cells/*μ*L of undiluted eluate.

DNA content and OD 260/280 ratios were determined using a spectrophotometer (NanoDrop 1000 Spectrophotometer version 3.7.1; NanoDrop Technologies, Wilmington, DE) per manufacturer's instructions.

### 2.3. Chlamydiaceae Diagnostic qPCR and Internal Amplification Control

23S rDNA gene-based Chlamydiaceae family-specific qPCR was carried out as previously described [3, 4]. In brief, DNA samples were evaluated on an ABI 7500 Fast Real-Time PCR System (Applied Biosystems, Foster City, CA, USA), using forward primer Ch23S-F (5′-CTGAAACCAGTAGCTTATAAG CGGT-3′), reverse primer Ch23S-R (5′-ACCTCGCCGTTTAACTTAACTCC-3′), and probe Ch23S-p (FAM-CTCATCA TGCAAAAGGCACGCCG-TAMRA) to amplify a 111 bp product specific for the Chlamydiaceae family.

The internal amplification control (177-bp product), which was included concomitantly with all samples, consisted of primers EGFP-1-F (5′-GACCACTACCAGCAGAACAC-3′) and EGFP-10-R (3′-CTTGTACAGCTCGTCCATGC-5′) and probe EGFP-HEX (HEX-AGCACCCAGTCCGCCCTGAGCA-BHQ1) (Intype IC-DNA, Qiagen Leipzig GmbH, Leipzig, Germany) [4, 5].

Quantities per 25 *μ*L reaction were as follows: 2.5 *μ*L of extracted DNA, 12.5 *μ*L of Mastermix (TaqMan Fast Universal PCR Mastermix (2x), no AmpErase UNG, Life Technologies, Carlsbad, CA), and a final concentration of 5 pmol/*μ*L of each primer and the probe (Microsynth, Balgach, Switzerland). The cycling program was as follows: initial denaturation (95°C, 20 seconds) followed by 45 cycles of amplification (95°C, 3 seconds; 60°C, 30 seconds), with an automatically calculated cycle threshold value. A Ct value of >38, in the case of Chlamydiaceae assay, was considered to be a negative result. A Ct value of >32 was considered to be negative for the internal amplification control target [6].

### 2.4. 18S rRNA qPCR

A commercially available endogenous control for eukaryotic 18S rRNA was used (Eukaryotic 18S rRNA Endogenous Control (FAM/MGB probe, non-primer limited, Life Technologies)). Analysis was carried out using the above-described PCR Mastermix, as recommended for our qPCR instrument, using the denaturation and amplification conditions and number of cycles as described above for Chlamydiaceae diagnostic qPCR. Volumes per 25 *μ*L reaction were as follows: 12.5 *μ*L 2x Mastermix, 1.25 *μ*L 20x 18S probe/primers, and 2.5 *μ*L sample. The threshold value was automatically calculated and Ct values of <38 were considered positive. This positivity cutoff was determined based on serial dilutions generated from HeLa cell DNA extracted via both described methods.

### 2.5. Samples

Chlamydiaceae diagnostic samples representative of the range of animals and sample types evaluated in our laboratory were chosen for DNA extraction. In detail, they consisted of material collected from turtle (liver/lung/spleen pool, FFPE material), guinea pig (uterus and eye, flocked swab material), alpaca (placenta, fresh organ material), pig (lung and spleen, fresh), and pig (eye and rectum, swabs). Additionally, DNA extracts were generated from chinchilla, chicken, cat, dog, mouse, guinea pig, and turtle liver FFPE material.

## 3. Results and Discussion

The first set of samples evaluated in this study (see Table 1) consisted of archived DNA extracted from fresh tissue samples stored at −20°C, swabs stored at −20°C, or FFPE tissues taken from various animals. These samples were collected during 2013 for routine Chlamydiaceae diagnostic evaluation. Samples representative of the range of animals and sample types evaluated in our laboratory were chosen as follows: FFPE block containing turtle liver/lung/spleen (single multiorgan sample), guinea pig uterus swab and eye swab (two swabs taken from one animal, at necropsy), fresh goat liver and lung samples (one multiorgan sample from each of two animals), fresh alpaca placenta sample, fresh swine lung sample and spleen sample (two single-organ samples from a single animal), and one each of eye and rectal swab samples from a single swine. The samples were evaluated using 23S rDNA gene-based Chlamydiaceae family-specific qPCR as previously described [3], and all were found to be Chlamydiaceae negative. During this initial diagnostic qPCR, an internal amplification control was routinely added to the reaction, as previously described [4, 5]. This target control DNA will be amplified in the absence of potential qPCR inhibitors present in the sample and its amplification is thus an indicator for a functioning reaction milieu.

**Figure 1:**
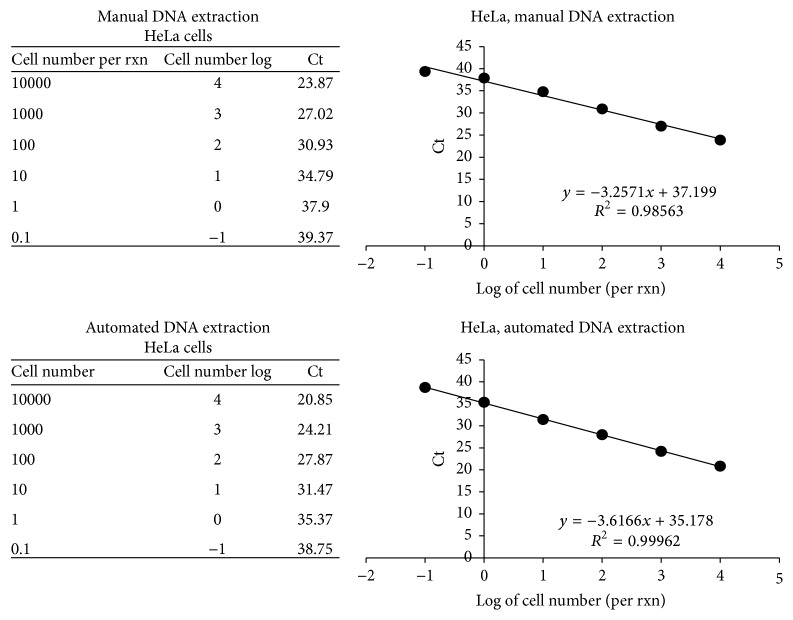
18S rRNA qPCR, serially diluted HeLa DNA.

For this study, a commercially available endogenous control for eukaryotic 18S rRNA, typically employed to allow quantitation of relative gene expression in complementary DNA samples, was used to determine the presence of amplifiable 18S rRNA gene in these various sample types. A positivity cutoff was determined based on serial dilutions made from HeLa cell DNA extracted via both automated and manual methods, wherein samples corresponding to DNA extracted (by either method) from at least 1–10 cells were considered positive, and samples corresponding to diluted DNA representative of <1 cell were considered negative (see Figure 1). The sample DNA concentrations and mean cycle threshold results (generated from duplicate wells) are shown in Table 1. All DNA concentrations were at least 12.5 ng/*μ*L, corresponding to at least 31.25 ng/reaction. All OD 260/280 ratios were between 1.6 and 1.9 with an average of 1.8 (standard deviation = 0.09, *n* = 10). All samples tested, regardless of animal species, tissue type, or swab sample, with the exception of a single swine rectal swab, showed 18S rRNA gene amplification by less than 23 cycles.

The representative range of animals, tissues, and swab samples commonly submitted to our laboratory for diagnosis of Chlamydiaceae was selected specifically to confirm that the described 18S rRNA gene qPCR is appropriate/sufficient to confirm that Chlamydiaceae negative samples contain amplifiable host DNA for a wide range of animal species. The DNA from the majority of these samples was extracted using automated equipment and the associated extraction kit. The less commonly encountered turtle sample was included to confirm that a nonmammalian 18S rRNA gene is sufficiently conserved to allow amplification. Additionally, the turtle sample was an FFPE sample and the DNA was extracted manually with an extraction kit commonly used for research purposes. Our findings confirm that the 18S rRNA gene is an appropriate target for demonstration of eukaryotic DNA content in a variety of sample species, tissues, and alternative sample types (swabs), as expected based on the wide-ranging conservation of 18S rRNA gene in eukaryotes.

Spectrophotometry (e.g., by NanoDrop method) alone can ensure that nucleic acids are present and has value to quickly show if technical errors have resulted in insufficient DNA extraction and if DNA is relatively pure, but it cannot be used to differentiate species of DNA, single stranded DNA, or single nucleotides from more intact genomic DNA. Because the amplicon size generated in this assay is 187 base pairs (per manufacturer's information), the assay gives a relatively rapid confirmation, compared to gel electrophoresis analysis, that excessive DNA fragmentation of a sample has not occurred, at least for the purposes of the described Chlamydiaceae diagnostic qPCR [3], which results in 111-base-pair product. For downstream applications requiring larger intact sections of DNA, size of the amplicon intended for use as a control as described herein must be considered accordingly.

The swine rectal swab sample, though 18S rRNA gene-negative, contained 130.8 ng/*μ*L DNA, effectively eliminating the possibility that DNA was not extracted due to technical error. Rectal swabs are commonly contaminated with feces, which contain various compounds and molecules, such as complex polysaccharides, known to inhibit PCR. Feces or feces-contaminated samples, as such, are considered to be potentially difficult to utilize for DNA extraction and PCR analysis [7]. Because the sample was verified to lack inhibitors of PCR amplification by successful amplification of the internal amplification control, we are confident that such inhibition did not result in the negative result. The OD 260/280 ratio for the sample was 1.6, the lowest such value within the group of samples being evaluated, the remaining of which had values of at least 1.8. However, this value is representative of those frequently recorded in our laboratory for research and/or diagnostic swine rectal swab samples (positive or negative for Chlamydiaceae), and often rectal or eye swabs may be the only samples available for evaluation.

We expect that this swine rectal swab sample contained relatively high levels of bacterial DNA and/or degraded swine DNA. This might occur in a sample likely to contain substantial fecal contamination, as feces harbor very high bacterial loads, and bacterial DNases may degrade DNA [8]. For the purposes of veterinary chlamydial diagnostics, because these bacteria may be shed in the feces, feces-contaminated samples, even if they lack sufficient host cell epithelial cells/DNA, or even feces samples specifically, are not precluded from diagnostic evaluation. However, the described 18S rRNA gene qPCR analysis may not be optimal in determining the quality of fecal samples or heavily feces-contaminated rectal swabs because DNA samples consisting largely or entirely of prokaryotic DNA may still prove useful for further molecular analysis, depending on the intended target(s). The results for these samples illustrate a method for improved determination of sample integrity by inclusion of 18S rRNA gene qPCR analysis in addition to spectrophotometric analysis and internal controls to evaluate the presence of PCR inhibitors. Future work evaluating Chlamydiaceae family-specific qPCR positive and negative rectal swabs will help clarify which control measures might improve quality validation for these types of samples.

Although the single FFPE sample included in the above evaluation proved to be positive for the eukaryotic 18S rRNA gene, FFPE specimens in general are well known to be less desirable for DNA extraction and analysis due to DNA fragmentation [1]. Therefore we evaluated an additional group of FFPE samples specifically to ensure that this sample type yielded consistent amplification of the eukaryotic 18S rRNA gene. Additionally, in our experience, the manual DNA extraction method frequently results in higher cycle thresholds when equivalent samples were processed for various qPCR analyses, even when higher DNA yield is achieved, compared to the automated method. Therefore, both DNA extraction methods routinely used for diagnostic and research purposes in our laboratory were compared for the purpose of ensuring that the manual method did not impact the usefulness of the 18S rRNA gene qPCR for sample quality control.

This second set of samples (see Table 2) consisted of FFPE liver samples from several animal species (chinchilla, chicken, dog, cat, mouse, guinea pig, and turtle). We used the same experimental protocol, described above for the first set of samples, to verify presence of amplifiable 18S rRNA gene in these FFPE liver samples. The DNA concentrations and mean cycle threshold results (generated from duplicate wells) are shown in Table 2. All DNA concentrations were at least 8.9 ng/*μ*L, corresponding to at least 22.25 ng/reaction. Notably, the automated DNA extraction method OD 260/280 ratios, ranging from 1.7 to 1.8 with an average of 1.7 (standard deviation = 0.05, *n* = 7), were consistently lower than those of the manual DNA extraction method, which ranged from 1.7 to 2.0 with an average of 1.9 (standard deviation = 0.11, *n* = 7). All samples tested, regardless of animal species or extraction method, showed 18S rRNA gene amplification by less than 29 cycles.

The automated DNA extraction method frequently resulted in greater DNA yield from equivalent samples, and in all cases, regardless of DNA yield, resulted in lower mean cycle thresholds than the corresponding manual DNA extraction method. Interestingly, this was true despite the association of the automated DNA extraction method with lower OD260/280 ratios. However, for all samples examined herein, both extraction methods are sufficient to allow 18S rRNA gene amplification and may be regarded as equivalent in the context of the Ct <38 positivity cutoff. The analysis of turtle and chicken samples again verifies that the 18S rRNA gene target is appropriate for analysis of samples from a wide range of animals that might be evaluated for the presence of Chlamydiaceae. Furthermore, we demonstrate here that FFPE samples from various animals processed by standard methods and submitted to frequently used DNA extraction methods consistently yield DNA of sufficient fragment length/quality to allow demonstration of presence of the 18S rRNA gene by qPCR. In cases for which DNA is extracted with Gram-positive bacteria as a target, a bead-beating step is routinely recommended in the DNA extraction process to break down the bacterial wall. We did not evaluate this extraction method in the current study but would not expect this step to interfere with the successful extraction of host 18S DNA. Specific extraction methods should be evaluated, depending on target DNA (Gram-positive versus Gram-negative bacteria, other microorganisms, etc.).

The need for quality control measures in molecular diagnostics is well accepted, and for veterinary applications the amplification of reference genes for such controls is complicated by the wide range of animal species that may be encountered. A previous report evaluated the proliferative cell nuclear antigen gene for use as such a control and was successful in amplification from a wide range of fresh and FFPE mammalian tissues [9]. The current study additionally considers avian and reptile samples, swab samples from various sites, and multiple commonly used DNA extraction methods to evaluate a wider range of sample types and animal species.

## 4. Conclusions

Due not only to the high level of conservation of the 18S rRNA gene amongst eukaryotes [10] but also to the presence of multiple specific variable regions [11], 18S rRNA-targeting primers can be designed for a variety of purposes to evaluate sample quality or determine species represented in a sample. For our purposes, we assessed the usefulness of an rRNA gene primer/probe set for confirming host DNA in a variety of sample types commonly evaluated in our laboratory for the presence of Chlamydiaceae, both for routine diagnostics and during the course of research studies. We propose that the application of this primer/probe set may be useful to verify quality of DNA samples of various animal, tissue, and sample types, including some swab specimens and FFPE specimens, for qPCR diagnoses of various microbial agent targets.

Another application for the described 18S rRNA qPCR could be the preliminary screening of archived samples prior to acceptance for biobanking, the need for which is highlighted by the fact that biobank projects have received sets of samples of which only a small fraction was actually suitable for the intended research (https://www.genomeweb.com/pcrsample-prep/betting-bank). FFPE specimens, extensively archived in pathology and histology departments and commonly used in all areas of biomedical research, have been generated and stored for many years [1]. And though these sample types are particularly noted to have reduced quality for nucleic acid analysis, interest in the continued use of FFPE specimens has prompted much consideration of ways to optimize processing, storage, and preanalytic quality control measures to improve the long term utility of these samples [12, 13].

## Figures and Tables

**Table 1:** Chlamydiaceae negative samples, 18S rRNA qPCR.

Animal	Sample type	DNA (ng/*µ*L)	OD 260/280^*∗∗*^	Mean Ct^*∗*^
Alpaca	Placenta	12.5	1.8	21.94

Goat	Liver and lung	188.3	1.9	15.48

Goat	Liver and lung	22.7	1.8	22.89

Guinea pig^†^	Uterus swab	140.7	1.9	20.72
	Eye swab	307.4	1.9	18.41

Swine	Lung	304.3	1.8	14.44
	Spleen	274.3	1.9	14.90

Swine	Eye swab	1118.4	1.9	14.39
	Rectal swab	130.8	1.6	#

Turtle	Liver, lung, spleen^§^	256.3^‡^	1.9	17.43

^*∗∗*^OD 260/280: absorbance at 260 nm/absorbance at 280 nm.

^*∗*^Mean Ct: mean cycle threshold, generated from duplicate wells.

^†^Swabs were taken at necropsy.

^‡^Manual/kit extracted DNA; all other samples were automated/kit extracted.

^§^FFPE sample; all other samples were taken from fresh tissues.

^#^Undetected.

**Table 2:** Formalin-fixed paraffin-embedded liver samples, 18S rRNA qPCR.

Animal	DNA extraction method					
	Manual/kit			Automated/kit		
	DNA (ng/*µ*L)	OD 260/280^*∗∗*^	Mean Ct^*∗*^	DNA (ng/*µ*L)	OD 260/280^*∗∗*^	Mean Ct^*∗*^
Chinchilla	47.1	1.7	28.24	124.8	1.7	25.91
Chicken	49.1	1.9	27.83	78.2	1.8	25.32
Dog	195.7	1.9	22.44	139.9	1.7	20.38
Cat	219.9	2.0	21.42	128.7	1.7	18.84
Mouse	97.8	2.0	27.54	82.6	1.8	24.40
Guinea pig	63.1	1.9	23.74	125.0	1.8	21.93
Turtle	8.9	1.8	28.06	16.4	1.8	25.83

^*∗∗*^OD 260/280: absorbance at 260 nm/absorbance at 280 nm.

^*∗*^Mean Ct: mean cycle threshold, generated from duplicate wells.
